# Effect of distributed Bragg reflectors on photoluminescence properties of CH_3_NH_3_PbI_3_ film

**DOI:** 10.1038/s41598-022-14991-4

**Published:** 2022-06-29

**Authors:** Feng Jiang, Zhiguang Xiao, Mengqi Dong, Jiawen Song, Yinong Wang

**Affiliations:** 1 Faculty of Electronic Information and Electrical Engineering, Dalian University of Technology, Dalian, 116024 People’s Republic of China; 2 Leicester International Institute, Dalian University of Technology, 2 Dagong Road, Liaodongwan, New District, Panjin, 124221 People’s Republic of China; 3 School of Physics, Dalian University of Technology, Dalian, 116024 People’s Republic of China

**Keywords:** Nanoscience and technology, Materials science

## Abstract

The nanoporous (NP) GaN distributed Bragg reflector (DBR) was prepared by using electrochemical etching. Then the NP-GaN DBR was pretreated by using ozone treatment. Atomic force microscopy and X-ray diffraction (XRD) were used to investigate the influence of ozone treatment on the structure of the substrates. The hybrid organic–inorganic CH_3_NH_3_PbI_3_ perovskite films were grown on the NP-GaN DBR and reference substrates by using a one-step solution method. The XRD and field emission scanning electron microscopy test results indicate the high quality of the prepared CH_3_NH_3_PbI_3_ perovskite films. The photoluminescence intensity of the prepared CH_3_NH_3_PbI_3_ perovskite film on the NP-GaN DBR substrate is ~ 3.5 times higher than that of the film on the reference substrate, with a 3.6 nm spectral blue-shift. The enhancement should be contributable to amplify spontaneous emission by resonant cavity, while the blue-shift could be contributable to stoichiometric difference of the films on different substrates.

## Introduction

The hybrid organic–inorganic perovskite materials have attracted considerable attention due to their excellent photoelectric properties^1–3^. In addition to being used in photovoltaic devices, perovskite materials have another feature which is luminescence through charge combination. Due to the direct and adjustable bandgap, perovskite materials have excellent application prospects in the field of photoelectric display, such as laser and light emitting diode^4–6^. As a kind of photoelectric material, how to improve the luminescent efficiency of perovskite is a hot topic of concern. At present, the luminescent efficiency is mostly improved by changing the chemical composition or structure of perovskite materials^6,7^.

Distributed Bragg reflector (DBR) is a kind of optical device which can enhance the reflection of light at different wavelengths using “constructive interference” of reflected lights at different interfaces^8–10^. Compared with the traditional Al or Ag mirrors which can cover the whole wave band from near ultraviolet to near infrared, DBR can selectively adjust the reflective wavelengths by setting the refractive index or thickness of different layers. Therefore, DBR has been used to prepare lasers with high monochromaticity^11^. On the other hand, Al or Ag mirrors are hydrophilic, so they are difficult to be used as the substrate of hybrid organic–inorganic perovskite materials. The structure of DBR is a periodic stack of films with different refractive indexes. When light passes through these periodic films, the reflected lights at different interfaces will interfere to enhance the reflected light. In recent years, various DBRs such as (Ga, Al, In)N/GaN, GaAs/AlAs, and nanoporous (NP) GaN/GaN periodic structures have been extensively researched^10,12–15^. Among them, inorganic perovskite (e.g. CsSnBr_1.8_I_1.2_) films have been embedded on the NP-GaN DBR, which has 4.3-fold luminescent efficiency higher than the reference film^10^. The above-mentioned DBRs are inorganic non-metallic materials, which are mostly hydrophilic rather than lipophilic. Therefore, hybrid organic–inorganic perovskite materials which are usually dissolved in *N*,*N*-dimethylformamide (DMF) and dimethyl sulfoxide (DMSO) are difficult to be embedded on the above DBRs by the most frequently used spin-coating method.

In this work, in order to make the hybrid organic–inorganic perovskite film grow on the NP-GaN DBR smoothly, the NP-GaN DBR and unetched GaN substrates were pretreated by ozone treatment. Then the hybrid organic–inorganic CH_3_NH_3_PbI_3_ perovskite films were prepared by using a one-step solution method on the above-mentioned substrates. The photoluminescence (PL) intensity of CH_3_NH_3_PbI_3_ film grown on the NP-GaN DBR is ~ 3.5-fold enhanced compared to that of the film grown on the unetched GaN substrate. This work demonstrates that the NP-GaN DBR can be a large-area platform for enhancing the PL quantum yield of hybrid organic–inorganic perovskite materials. And it presents a new practical and effective method of designing perovskite devices for broadband and large-area application, such as solar cells, vertical-cavity surface emitting lasers, and light-emitting diodes.

## Materials and methods

### The NP-GaN DBR fabrication

**Figure 1:**
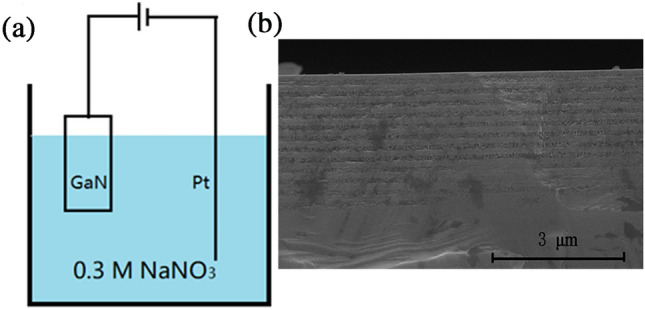
(**a**) Schematic diagram of the EC etching setup. (**b**) Cross-sectional image of GaN-based sample after the etching measured by scanning electron microscopy.

Electrochemical (EC) etching method was carried out in a two-electrode cell under room light and temperature. Ga-polar GaN sample and platinum wire were used as anode and cathode electrodes, respectively. Here, the GaN epitaxial film, which was grown on c-plane sapphire substrate with a low-temperature GaN buffers by metal–organic chemical vapor deposition, was a 12-pair undoped GaN (u-GaN)/Si-doped GaN (n-GaN). The thicknesses of u-GaN and n-GaN layers were 70 and 95 nm, respectively. The doping concentration of n-GaN layers was 1 × 10^19^ cm^−3^. The EC etching was carried out in 0.3 mol/L NaNO_3_ aqueous solution at an applied bias of 9 V. Figure 1a shows the schematic diagram of the EC etching setup. After the etching, GaN samples were rinsed with deionized water and dried in N_2_. The GaN sample presented GaN/NP-GaN periodical structure (Fig. 1b). As a reference, a 8 × 10^18^ cm^−3^ Si-doped GaN film with a thickness of 2 μm on c-plane sapphire substrate was etched in 0.3 mol/L NaNO_3_ aqueous solution at applied bias of 15 V for 3 min.

### The perovskite precursor solution preparation

The precursor solution was mixed with 1.1 M Methylamine iodide (CH_3_NH_3_I, 99.5%, Bouletta), 1 M Lead iodide (PbI_2_, 99.999%, Aldrich) in 1 mL DMF (> 99.8%, Aldrich) and DMSO (> 99.7%, Aldrich) (the volume ratio of DMF:DMSO is 7:3) at room temperature and stirred for 6 h, until the pale yellow precursor solution was observed. All chemicals were used as received without further purification.

### The CH_3_NH_3_PbI_3_ perovskite film fabrication

The NP-GaN DBR was wiped with ethyl alcohol several times and then transferred to a UV-O_3_ chamber for 15 min. The treated NP-GaN DBR substrate was placed on the hot plate at 50 °C for 15 min. Then 45 μL precursor solution was spin-casted onto the NP-GaN DBR at 1000 rpm for 12 s and 4000 rpm for 45 s, and then annealed at 100 °C for 20 min. Moreover, 500 μL isopropanol (99.5%, Sinopharm) was dropped on the substrate 15 s before the end of the procedure. The same operations were implemented on the unetched and etched single-layer GaN substrates. All of the above operations were performed in the air environment.

### Structural and optical characterization

The surface morphology of the NP-GaN DBR untreated and treated by ozone was investigated by using atomic force microscopy (AFM, Dimension Icon). The X-ray diffraction (Lab XRD-7000s, Shimadzu) was employed to determine the structural properties of the prepared films with angular range of 10° ≤ 2θ ≤ 70° via Cu *K*α radiation (λ = 1.5405 Å), operating at 40 kV and 30 mA. The morphology of perovskite films was acquired on a field emission scanning electron microscopy (Nova Nano SEM 450, FEI). The reflectance spectra of the etched samples were measured by UV–Visible spectrophotometer (Hitachi, U-4100). The PL measurements of the prepared films were acquired by using Hitachi F-7000 fluorescence spectrometer. All of the prepared films were photoexcited using a 517 nm laser.

## Results and discussions

**Figure 2:**
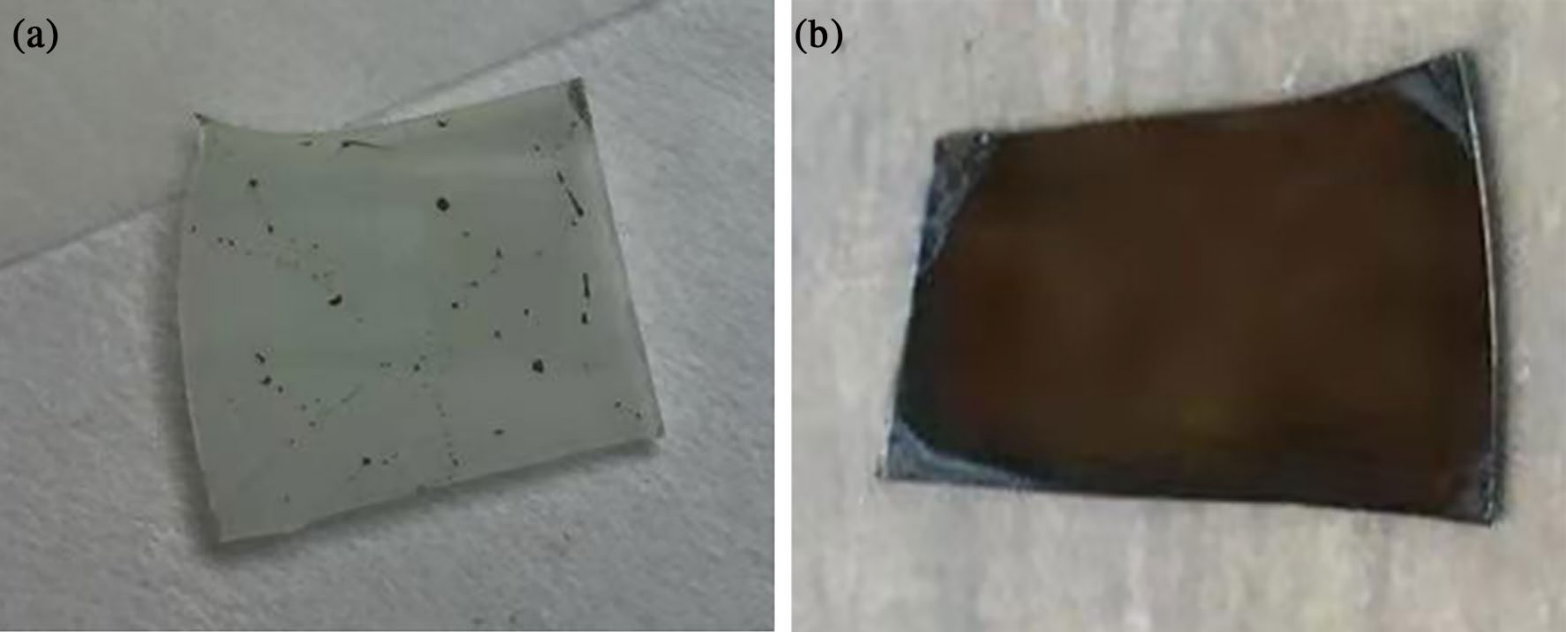
Top-view optical images of the perovskite films grown on the NP-GaN DBRs untreated (**a**) and treated (**b**) by ozone.

Figure 2a,b show the top-view optical images of the perovskite films grown on the NP-GaN DBRs untreated and treated by ozone. The image confirms the fact that continuous hybrid organic–inorganic CH_3_NH_3_PbI_3_ perovskite film can not grow on the NP-GaN DBR without any treatment (Fig. 2a), indicating that Ga-polar GaN surface is not lipophilic. To grow continuous CH_3_NH_3_PbI_3_ films, Ga-polar GaN substrate was treated by ozone. It can be observed that a continuous, uniform perovskite film was grown on the ozone treated NP-GaN DBR, as shown in Fig. 2b.

**Figure 3:**
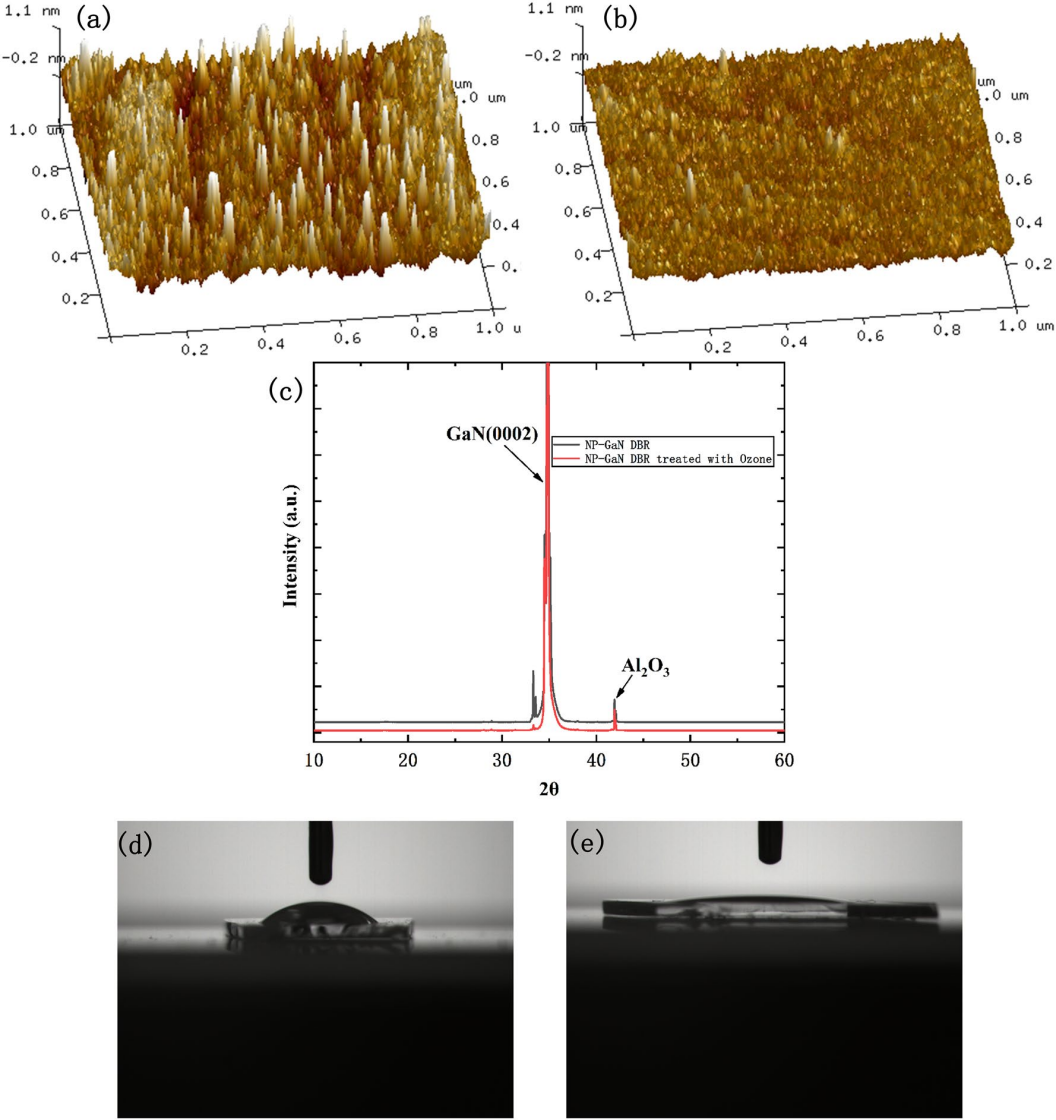
The AFM surface micrograph of the NP-GaN DBRs (**a**) untreated and (**b**) treated by ozone. (**c**) The XRD patterns of the NP-GaN DBRs untreated and treated by ozone. The contact angles between the NP-GaN DBR and the precursor solution (**d**) untreated and (**e**) treated by ozone.

To elucidate the ozone modification mechanism of GaN surface, the surface of Ga-polar NP-GaN DBR was investigated. Figure 3a,b show the AFM surface micrographs of the NP-GaN DBRs untreated and treated by ozone. The root mean square roughness of the NP-GaN DBR changes from 0.190 to 0.139 nm after ozone treatment, indicating that ozone treatment can lead to a smoother surface of the NP-GaN DBR. Figure 3c shows the XRD patterns of the NP-GaN DBRs treated and untreated by ozone. Ozone treatment has no essential influence on the chemical constitution of the NP-GaN DBR. In addition, the two peaks located at ~ 30.8° and ~ 37.0° which correspond to Ga (102) and Ga (211) planes are not observed in the XRD patterns. This demonstrates that the gallium droplets which have been seen in many GaN epitaxial wafers according to previous reports^16–18^, do not exist in the prepared NP-GaN DBR, implying that the EC etching can reduce the concentration of Ga in the film, leading to the high quality of the substrate. Figure 3d,e show the contact angles between the NP-GaN DBR and the precursor solution untreated and treated by ozone. The contact angle untreated by ozone is 36.6°. The ozone treatment enhances the surface activity, and the contact angle becomes 7.1°, meaning that the perovskite film can grow on the ozone treated NP-GaN DBR continuously.

**Figure 4:**
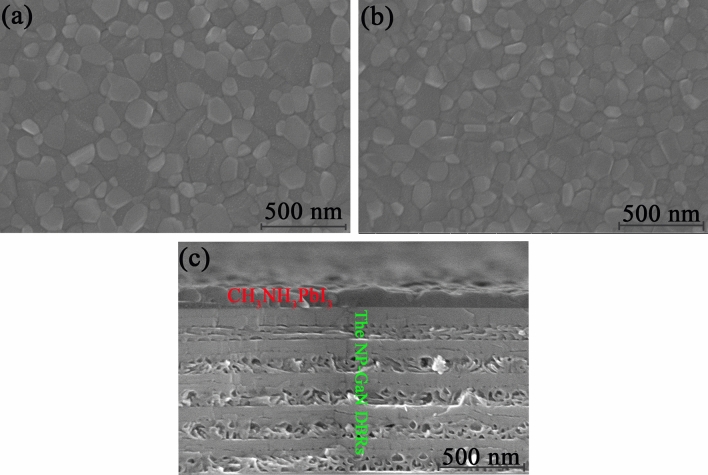
The top-view SEM morphology images of perovskite films grown on (**a**) the NP-GaN DBR and (**b**) as-grown GaN substrate by one-step solution method. (**c**) The cross-sectional SEM image of CH_3_NH_3_PbI_3_ perovskite thin film grown on the NP-GaN DBR.

Figure 4a,b show the top-view SEM morphology images of perovskite films grown on the NP-GaN DBR and as-grown GaN substrate. It can be seen that the prepared CH_3_NH_3_PbI_3_ films on both of the NP-GaN DBR and as-grown GaN substrate are homogeneous, highly-crystallized and compact. The two perovskite thin films grown on different GaN substrates have the same morphology which is monolithically grained, without any non-uniform crystalline structures such as dendrites. The grain sizes of the two films are in the range of 50–180 nm. The SEM images indicate a high crystal quality of the prepared perovskite thin films. Figure 4c shows the cross-sectional SEM image of CH_3_NH_3_PbI_3_ perovskite thin film grown on the NP-GaN DBR. The periodic structure of the NP-GaN DBR can be observed in Fig. 4c. It can be seen that the CH_3_NH_3_PbI_3_ perovskite thin film grown on the NP-GaN DBR is a void-free continuous film, with a thickness of ~ 110 nm.

**Figure 5:**
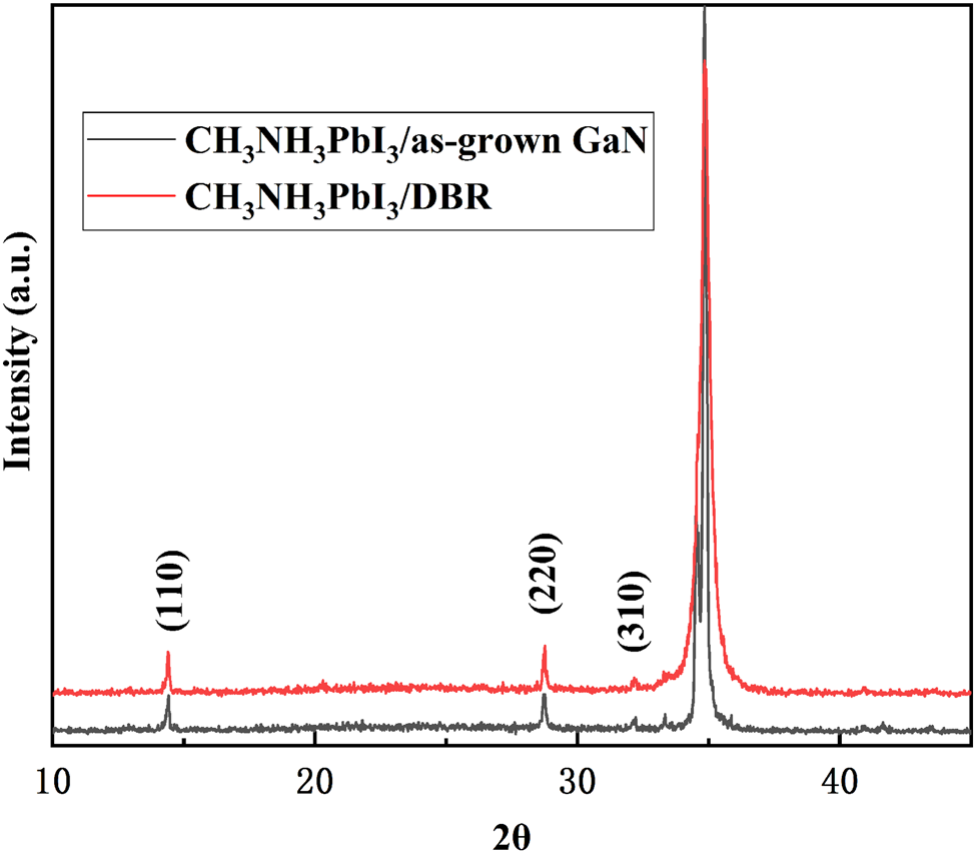
The XRD patterns of the perovskite thin films grown on the NP-GaN DBR and as-grown GaN substrate.

Figure 5 shows the XRD patterns of the perovskite thin films grown on the NP-GaN DBR and as-grown GaN substrate. The diffraction peaks at 14.4°, 28.8°, and 32.2°, which correspond to the (110), (220), (310) lattice planes of a tetragonal CH_3_NH_3_PbI_3_ structure reported previously^19^, state clearly the high crystallinity of the prepared film. The diffraction peaks at 33.4° and 34.9° are indexed to the NP-GaN DBR^20,21^. The diffraction peaks intensities of the prepared perovskite films are significantly smaller than that of the NP-GaN DBR, and the baseline is not straight enough. This is due to the fact that the CH_3_NH_3_PbI_3_ perovskite film is thin (only ~ 110 nm, as shown in the Fig. 4c) and it exists in the form of thin film instead of crystal.

**Figure 6:**
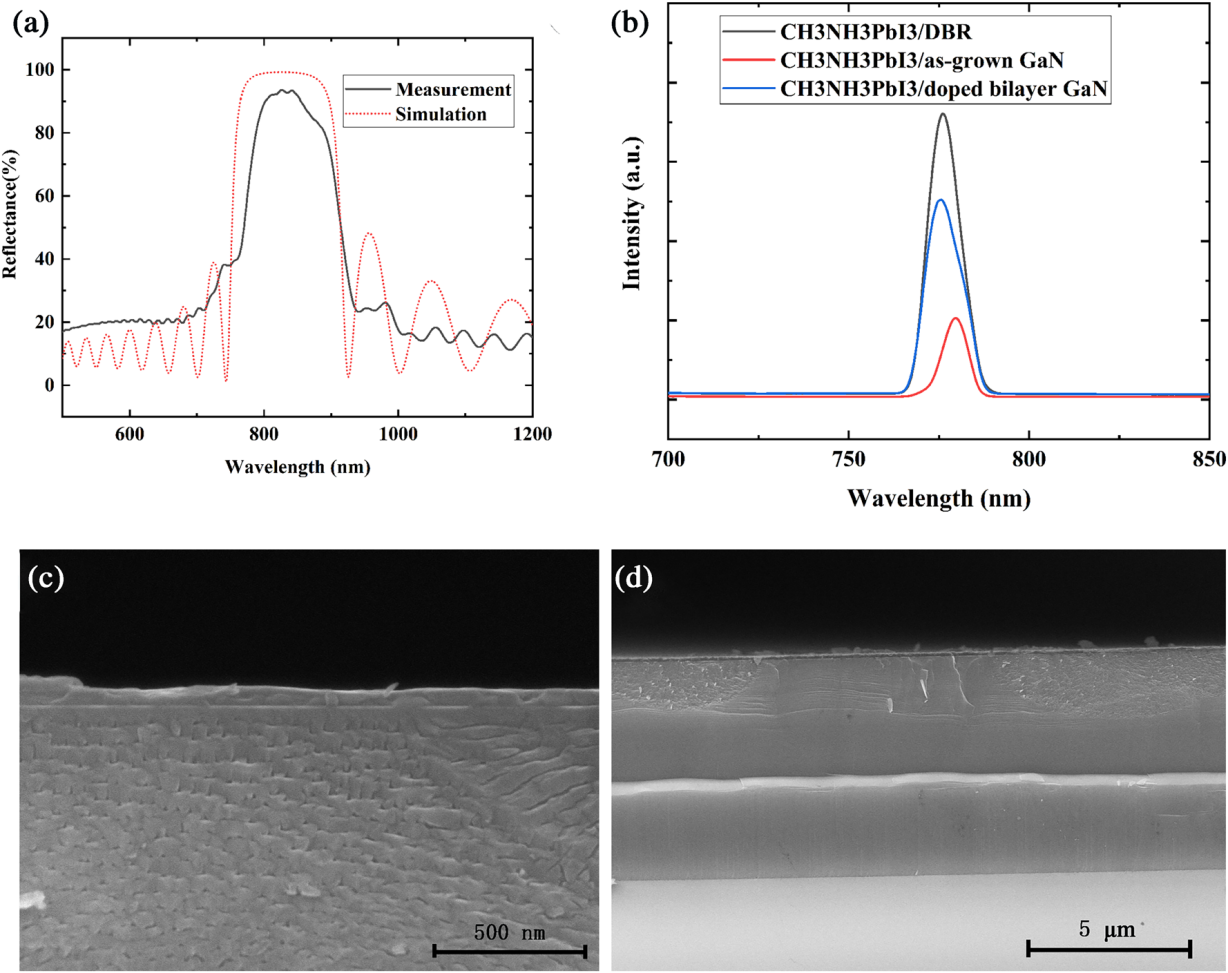
(**a**) The experimental and simulated reflectance spectra of the NP-GaN DBR. (**b**) The room-temperature PL spectra of the prepared perovskite films on the NP-GaN DBR, etched single-layer GaN and as-grown GaN substrates. (**c**,**d**) The cross-sectional SEM images of CH_3_NH_3_PbI_3_ perovskite thin film grown on the etched single-layer GaN substrate with different resolution.

Figure 6a shows experimental and simulated the reflectance spectra of the prepared NP-GaN DBR. According to the volume averaging theory^22^, the effective refractive index of the NP-GaN layer can be calculated by using the follow equation:

$$ n = \sqrt {(1 - \varphi )n^{2}_{GaN} + \varphi n^{2}_{air} } $$

where φ is the porosity in NP-GaN layers, n_GaN_ and n_air_ are refractive indexes of GaN and air. According to the cross-sectional SEM images in Fig. 4c, the porosity of u-GaN and n-GaN are ~ 5% and ~ 50%, respectively. According to the Essential Macleod database, the refractive index of bulk GaN is ~ 2.36 at 780 nm. The values of n_air_ is 1, so the refractive indexes of u-GaN and n-GaN layers are calculated to be 2.31 and 1.81, respectively. Based on the above-mentioned parameters, the NP-GaN DBR has a stop band from ~ 740 to ~ 920 nm with a peak reflectance of > 99%, according to the simulated reflectance spectrum shown in Fig. 6a. However, the experimental maximum reflectance is ~ 94%, whereas the reflectance is ~ 67% at 780 nm which is the emission wavelength of CH_3_NH_3_PbI_3_^5,23^. The results indicate that the EC etching is not uniform. Figure 6b shows the room-temperature PL spectra of the prepared perovskite films on the NP-GaN DBR, etched single-layer GaN and as-grown GaN substrates. The PL peak position which locates at about 780 nm is very closed to that of tetragonal CH_3_NH_3_PbI_3_ structure reported by Wong^5^. It can be seen that the PL intensity of perovskite film on the NP-GaN DBR is ~ 3.5 times higher than that of the film on the as-grown GaN substrate, while the value is ~ 2.5 times higher for the etched single-layer GaN substrate. As shown in Fig. 6c,d, the etched single-layer GaN substrate presents irregular nanopores, whereas the NP-GaN DBR with a reflectance of 67% at 780 nm is a 12-pair undoped GaN/Si-doped GaN substrate (Fig. 1b). The PL enhancement of the perovskite film grown the etched single-layer GaN can be contributable to scattering and reflection of nanopores, leading to amplify spontaneous emission (ASE) by resonant cavity^24^. The ASE occurs before the light output. The cavity is formed between the nanoporous GaN and CH_3_NH_3_PbI_3_ surface. Since the intensity of reflected and scattered light in the etched single-layer GaN is significantly lower than that of DBR interference light, the film on the NP-GaN DBR has the stronger PL intensity. On the other hand, the PL spectra of the perovskite films on the NP-GaN DBR and etched single-layer GaN substrate present about ~ 3.6 and ~ 4.5 nm spectral blue-shift, respectively. For both of the NP-GaN DBR and etched single-layer GaN substrate, the precursor will permeate into the hole of the etched top GaN layer during the spin-coating process. According to the previous report^25^, the 3D perovskites form a thin film starting from the solution surface as the solvent volatilizes rapidly. The solvent in the pore is hard to volatilize, resulting in a stoichiometric difference compared with the perovskite film grown on the unetched GaN substrate. And the Pb/I atomic ratios of perovskite films grown on NP-GaN DBR and etched single-layer GaN substrate measured by using energy dispersive spectrometer (EDS) are 0.346 and 0.363, respectively. Therefore, the blue-shift could be contributed to stoichiometric difference of the CH_3_NH_3_PbI_3_ films grown on different substrates^10,25–27^. As shown in Figs. 4c and 6c, the porosity of the top GaN layer for the NP-GaN DBR is much smaller than that on the etched single-layer GaN substrate. This results in a larger blue-shit of perovskite film on the etched single-layer GaN substrate. This PL intensity enhancement paves the way for designing and developing a range of perovskite devices for broadband and large-area application.

## Conclusion

In this work, NP-GaN DBR was fabricated by using electrochemical etching methods. The substrates were pretreated by ozone treatment. CH_3_NH_3_PbI_3_ perovskite films were grown on the DBR and reference substrates by using a one-step solution method to see the influence of DBR on the structure and optical properties of CH_3_NH_3_PbI_3_ films. The XRD and SEM results show that the CH_3_NH_3_PbI_3_ films can grow on the NP-GaN DBR continuously by ozone treatment. Compared to the reference substrate, the PL intensity of CH_3_NH_3_PbI_3_ film on the NP-GaN DBR can be enhanced significantly. This work provides a new method for enhancing the PL quantum yield of hybrid organic–inorganic perovskite materials, and it may be used in novel design of optoelectronic devices.

## References

[1] Kim HS (2012). Lead iodide perovskite sensitized all-solid-state submicron thin film mesoscopic solar cell with efficiency exceeding 9%. Sci. Rep..

[2] Liu MZ, Johnston MB, Snaith HJ (2013). Efficient planar heterojunction perovskite solar cells by vapour deposition. Nature.

[3] He Z (2021). Recent progress in metal sulfide-based electron transport layers in perovskite solar cells. Nanoscale.

[4] Zhu HM (2015). Lead halide perovskite nanowire lasers with low lasing thresholds and high quality factors. Nat. Mater..

[5] Wong AB (2015). Growth and anion exchange conversion of CH_3_NH_3_PbX_3_ nanorod arrays for light-emitting diodes. Nano Lett..

[6] Zhang F (2015). Brightly luminescent and color-tunable colloidal CH_3_NH_3_PbX_3_ (X = Br, I, Cl) quantum dots: Potential alternatives for display technology. ACS Nano.

[7] Levchuk I (2017). Ligand-assisted thickness tailoring of highly luminescent colloidal CH_3_NH_3_PbX_3_ (X = Br and I) perovskite nanoplatelets. Chem. Commun..

[8] Sharma R (2007). Gallium-nitride-based microcavity light-emitting diodes with air-gap distributed Bragg reflectors. Appl. Phys. Lett..

[9] Sharma R, Haberer ED, Meier C, Hu EL, Nakamura S (2005). Vertically oriented GaN-based air-gap distributed Bragg reflector structure fabricated using band-gap-selective photoelectrochemical etching. Appl. Phys. Lett..

[10] Liu J (2020). Fabrication and applications of wafer-scale nanoporous GaN near-infrared distributed Bragg reflectors. Opt. Mater..

[11] Chen S, Zhang C, Lee J, Han J, Nurmikko A (2017). High-Q, low-threshold monolithic perovskite thin-film vertical-cavity lasers. Adv. Mater..

[12] Wang GJ (2017). GaN/AlGaN ultraviolet light-emitting diode with an embedded porous-AlGaN distributed Bragg reflector. Appl. Phys. Express.

[13] Ng HM, Doppalapudi D, Iliopoulos E, Moustakas TD (1999). Distributed Bragg reflectors based on AlN/GaN multilayers. Appl. Phys. Lett..

[14] Gacevic Z (2010). InAlN/GaN Bragg reflectors grown by plasma-assisted molecular beam epitaxy. J. Appl. Phys..

[15] Ng HM, Moustakas TD, Chu SNG (2000). High reflectivity and broad bandwidth AlN/GaN distributed Bragg reflectors grown by molecular-beam epitaxy. Appl. Phys. Lett..

[16] Yang XK (2020). Fabrication and optoelectronic properties of Ga_2_O_3_/Eu epitaxial films on nanoporous GaN distributed Bragg reflectors. J. Mater. Sci..

[17] Feiler D, Williams RS, Talin AA, Yoon HJ, Goorsky MS (1997). Pulsed laser deposition of epitaxial AlN, GaN, and InN thin films on sapphire(0001). J. Cryst. Growth.

[18] Guo DY (2018). Self-powered ultraviolet photodetector with superhigh photoresponsivity (3.05 A/W) based on the GaN/Sn:Ga_2_O_3_ pn junction. ACS Nano.

[19] Zheng YZ (2017). Effects of precursor concentration and annealing temperature on CH_3_NH_3_PbI_3_ film crystallization and photovoltaic performance. J. Phys. Chem. Solids.

[20] Nyk M (2005). Structure and optical properties of MOVPE and HVPE GaN films grown on GaN nanocrystalline powder substrate. J. Cryst. Growth.

[21] Di Lello BC, Moura FJ, Solorzano IG (2002). Synthesis and characterization of GaN using gas-solid reactions. Mater. Sci. Eng. B Solid State Mater. Adv. Technol..

[22] Braun MM, Pilon L (2006). Effective optical properties of non-absorbing nanoporous thin films. Thin Solid Films.

[23] Abdy H (2019). Synthesis, optical characterization, and simulation of organo-metal halide perovskite materials. Optik.

[24] Su X (2020). Performance improvement of ultraviolet-A multiple quantum wells using a vertical oriented nanoporous GaN underlayer. Nanotechnology.

[25] Wang J (2020). Templated growth of oriented layered hybrid perovskites on 3D-like perovskites. Nat. Commun..

[26] Hakimian A, McWilliams S, Ignaszak A (2019). ZnO synthesized using bipolar electrochemistry: Structure and activity. Materials.

[27] Feng J, Yang XS, Li R, Yang XJ, Feng GW (2019). The Composition-dependent photoluminescence properties of non-stoichiometric ZnxAgyInS1.5+x+0.5y nanocrystals. Micromachines.

